# Altered Intrinsic Brain Activity and Functional Connectivity Before and After Knee Arthroplasty in the Elderly: A Resting-State fMRI Study

**DOI:** 10.3389/fneur.2020.556028

**Published:** 2020-09-29

**Authors:** Fei Lan, Guanwen Lin, Guanglei Cao, Zheng Li, Daqing Ma, Fangyan Liu, Mei Duan, Huiqun Fu, Wei Xiao, Zhigang Qi, Tianlong Wang

**Affiliations:** ^1^Department of Anesthesiology, Xuanwu Hospital, Capital Medical University, National Clinical Research Center for Geriatric Disorders, Beijing Institute for Brain Disorders, Beijing, China; ^2^Department of Anesthesiology, Hainan General Hospital, Hainan Affiliated Hospital of Hainan Medical University, Haikou, China; ^3^Department of Orthopedics, Xuanwu Hospital, Capital Medical University, Beijing, China; ^4^Department of Surgery and Cancer, Faculty of Medicine, Imperial College London, Anaesthesia Research of the Section of Anaesthetics, Pain Medicine and Intensive Care, Chelsea and Westminster Hospital, London, United Kingdom; ^5^Department of Radiology, Xuanwu Hospital, Capital Medical University, Beijing, China

**Keywords:** older patients, knee osteoarthritis, functional magnetic resonance imaging, amplitude of low-frequency fluctuation (ALFF), functional connectivity (FC), total knee arthoplasty

## Abstract

**Objective:** This study aimed to investigate the brain functional alterations with resting-state functional magnetic resonance imaging (rs-fMRI) in older patients with knee osteoarthritis (KOA) before and after total knee arthroplasty (TKA) and to assess the causal relationship of the brain function and neuropsychological changes.

**Methods:** We performed rs-fMRI to investigate brain function of 23 patients aged ≥65 with KOA and 23 healthy matched controls. Of the KOA patients, 15 completed postoperative rs-fMRI examinations. Analyzes of the amplitude of low-frequency fluctuation (ALFF) and functional connectivity (FC) were used to estimate differences in brain functional parameters between KOA patients, postoperative patients, and the controls. The relationship between changes of pre- and post-surgical status in ALFF and neuropsychological test results was analyzed.

**Results:** Compared with the controls, all patients with KOA exhibited decreased ALFF in the default mode network (bilateral angular gyrus, precuneus gyrus, medial superior frontal gyrus) and increased ALFF in the bilateral amygdala and cerebellum posterior lobe before surgery (*P* < 0.001). Altered ALFF persisted in the same brain regions 1 week postoperatively. The decreased ALFF in the left precuneus gyrus and middle temporal gyrus was found after surgery when compared with preoperative data (*P* < 0.01). Preoperatively, the KOA patients exhibited increased FC between the left precuneus gyrus and the right supplementary motor area compared to the controls (*P* < 0.001), but this connectivity became no significant difference after TKA. The left Cerebelum_9 was found to have decreased FC with the right precuneus gyrus postoperatively (*P* < 0.001) although this was not significantly different before surgery. The significantly altered ALFF values were not correlated with changes in cognitive assessment scores.

**Conclusion:** In older patients with end-stage KOA, functional alterations in important brain regions were detected with the persistence and further changes observed at an early stage after knee replacement. Our data further our understanding of brain functional abnormalities and cognitive impairment in older patients following knee replacement, which may provide therapeutic targets for preventive/treatment strategy to be developed.

**Trial registration:** Clinical Trial Registration: http://www.chictr.org.cn/index.aspx, ChiCTR1800016437; Registered June 1, 2018.

## Introduction

Knee osteoarthritis (KOA) is a common disease in elderly people worldwide; chronic pain and movement limitation caused by the disease seriously negate their quality of life (1). It has been suggested that KOA is associated with an increased risk of cognitive decline and dementia (2). Although the exact mechanism is still unclear (3), peripheral inflammatory mediators associated with osteoarthritis travel to the central nervous system to subsequently cause neuroinflammation and then trigger cognitive decline or even dementia/Alzheimer's disease development (4). Patients at end-stage KOA normally receive total knee arthroplasty (TKA), and surgery can relieve chronic pain through removal of diseased cartilage and synovium, which causes chronic peripheral inflammation. However, surgery can cause trauma and stress, all of which can further induce local and systemic inflammatory responses. Cognitive dysfunction can occur at the early stage after TKA (5, 6). Wide-ranging incidence of cognitive dysfunction has been reported in different patient populations, and it is likely related to the different type of neuropsychological test battery used, the variety of statistical analysis, and a lack of controls. In addition, the incidence of cognitive impairment can be overestimated or underestimated when the neuropsychological battery test itself is improperly used (7).

Brain imaging is relatively objective, and indeed, a neuroimaging study has shown that patients with KOA have structural brain alterations, especially in the prefrontal cortex, which is associated with cognitive decline compared with healthy controls (8). Lewis et al. (9) also report that region-specific gray matter atrophy was detected before surgery, and white matter integrity was improved after knee replacement. However, previous studies suggest that the functional brain alterations in cognitive impairment patients occurred prior to structural changes and actual manifestation of clinical symptoms (10). Nowadays, resting-state functional magnetic resonance imaging (rs-fMRI) is a popular tool for investigating neural mechanisms involved in various mental disorders (11–13). Also, it has been applied for uncovering functional brain changes in KOA patients (14, 15), which report that disturbance of connectivity in the default mode network (DMN) is commonly seen in these patients compared to healthy controls. Huang and colleagues (16) suggest that one quarter of older adults who undergo TKA under general anesthesia show a significant functional network decline within 48 h after surgery; however, a baseline preoperative functional network status was not done. Importantly, these studies only focus on using functional connectivity (FC) to investigate abnormal brain connections between two remote regions and do not evaluate abnormal regional brain activity. The amplitude of low-frequency fluctuation (ALFF), which is a quantitative measurement in rs-fMRI analysis, can provide information on the amplitude of regional spontaneous brain activity to indicate local brain functional abnormalities (17). No previous studies were performed to comprehensively analyze the type and extent of functional neuronal activity changes in patients with KOA before and at the early stage after TKA.

Given that the number of cases of KOA receiving TKA and showing cognitive impairment are increasing in an aging society, it is essential to know the integrated functional differences of the brain regions related to cognition in KOA patients before surgery and at the early stage after TKA compared to healthy matched controls. The aim of the current study is to investigate functional brain alterations in older patients with KOA by analyzing ALFF and FC to examine the further changes in brain function at the early stage after TKA and to determine the relationships between ALFF alterations and the neuropsychological changes following TKA.

## Materials and Methods

### Research Design and Population

**Figure 1:**
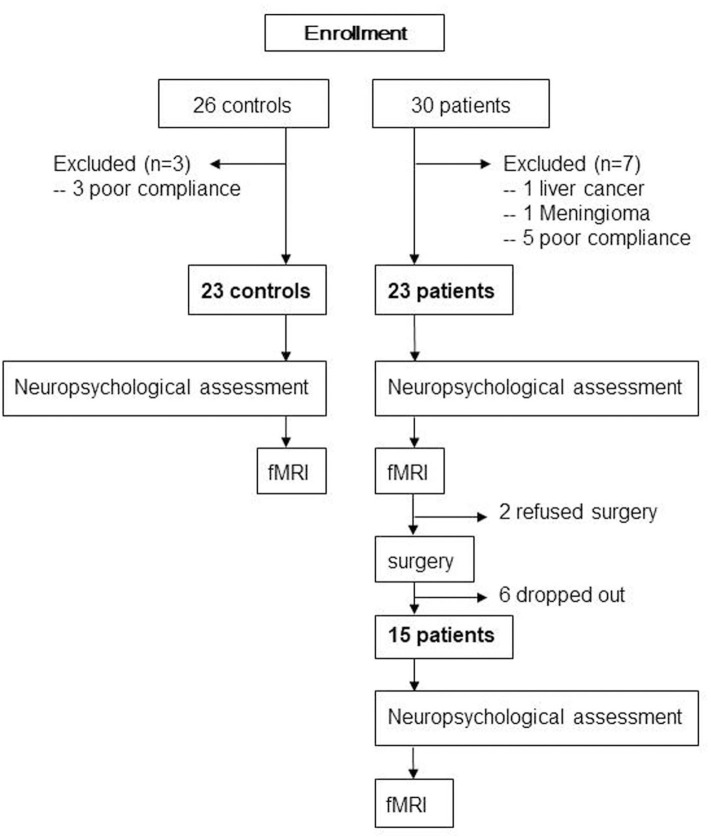
Flow diagram showing selection of eligible participants and the enrollment process.

The protocol was approved by the institutional review board of Xuanwu Hospital, Capital Medical University (Code: 2018-047), and then registered on the Chinese clinical trial registration (ChiCTR1800016437). After obtaining written informed consent in accordance with the Declaration of Helsinki, 30 patients aged ≥65 years with KOA who had intact daily living activities and underwent TKA between August 2018 and July 2019 at our institute and 26 matched controls were screened for recruitment (Figure 1). The diagnosis of KOA was made from medical history and imaging. All patients were assessed with the self-reported questionnaire in Chinese translated from the Western Ontario and McMaster Universities Osteoarthritis Index (WOMAC). The exclusion criteria were left-handed, baseline neuropsychological testing indicating dementia, any other major surgery within the research timeline, a history of head trauma/neurodegenerative illness, documented learning or seizure disorder, <6 years education, substance abuse, major cardiac disease, chronic medical illness known to induce encephalopathy, an implantable device precluding MRI, and unwillingness to complete MRI examinations. Two neuropsychologists reviewed the baseline data to confirm that test scores met the expected ranges for nondemented individuals. The healthy matched controls, who were age-, gender-, and education-matched with our patient cohort and had an absence of pain anywhere, were recruited through advertisements from the local community. Exclusion criteria for control subjects were history of neurologic, psychiatric, or any trauma that could have interfered with brain function and history of persistent knee pain.

### Anesthesia and Surgery

All patients received spinal anesthesia through a median or para-median approach using a 26 or 27 G needle with 2.0 ml 0.5% bupivacaine at the L3/4, and no intravenous sedatives were administered throughout. TKA was performed in a standard manner for all patients by the same surgeon group. A tourniquet was applied during surgery set to 250 mmHg during surgery and deflated just before wound closure. All patients received local infiltration anesthesia of a total of 100 ml 0.2% ropivacaine and 0.5 mg adrenaline in the surgical cutting area before incision closure. Upon completion of the surgery, all patients received a multimodal pain relief regimen postoperatively, including oral acetaminophen 1,000 mg and oral celecoxib 200 mg every 12 h. In addition, a continuous femoral nerve block for 3 days used a 20-ml bolus injection of 0.2% ropivacaine followed by a continuous infusion of 6 ml/h. A bolus injection of 6 ml was given per patient as needed. Furthermore, a single injection of subgluteal sciatic nerve block was administered with 25 ml of 0.2% ropivacaine once immediately after surgery.

### Neuropsychological Assessment

Baseline general cognitive data were collected from all participants using the Montreal Cognitive Assessment (MoCA) system, and then detailed neuropsychological tests were carried out by the same neuropsychologist. The tests included the verbal fluency test, auditory verbal learning test (AVLT; short-term and long-delayed-term recall), shape trails test–B, and clock drawing test (30 points in total). Particular attention was paid to memory and executive ability, which are commonly seen to decline after TKA (16, 18). All healthy matched controls underwent these assessments once, and all 23 patients received these assessments on the day just prior to MRI scan before surgery. Due to drop off or refused surgery, 15 of those patients had the assessment 1 week after surgery (Figure 1).

### Magnetic Resonance Image Data Acquisition

All 23 healthy matched controls received fMRI scan once; 23 patients had fMRI scan 1 day before surgery. Due to drop off or refused surgery, only 15 patients had fMRI 7 days after surgery (Figure 1).

MRI data were obtained using a Clinical 3 Tesla whole body MR imager (Verio; Siemens Medical Solutions, Erlangen, Germany) with a 32-channel head coil. The head coil was fitted with foam padding and headphones to minimize head motion and reduce scanner noise. During scanning, participants were asked to hold their head still, relax with their eyes closed, and try not to think of anything in particular. rs-fMRI images were generated using a rapid-gradient echo-planar imaging sequence (239 volumes, repetition time = 2000 ms, echo time = 40 ms, field of view = 240 × 240 mm^2^, flip angle = 90°, section thickness = 4 mm, acquisition matrix = 64 × 64, a total of 28 slices covering the whole brain). Three-dimensional T1-weighted magnetization-prepared rapid-gradient echo sagittal images were collected using the following parameters: repetition time = 1900 ms, echo time = 2.2 ms, inversion time (TI) = 900 ms, FA = 9°, resolution = 256 × 256 matrix, a total of 176 slices with a thickness of 1.0 mm, and voxel size = 1 × 1 × 1 mm.

### Individual-Level rs-fMRI Data Processing

Data processing was performed using the Configurable Pipeline for Analysis of Connectomes (C-PAC, https://fcp-indi.github.com), which is a python-based pipeline tool involving AFNI, ANTs, FSL, and custom python code. The whole analysis was accelerated and simplified through a cloud platform (http://www.humanbrain.cn, Beijing Intelligent Brain Cloud, Inc).

### Structural Processing

Structural processing included the following steps: (1) Images were de-obliqued; (2) images were reoriented into right-to-left, posterior-to-anterior, inferior-to-superior orientation; (3) skull stripping was performed; (4) individual skull-stripped brains were normalized to Montreal Neurological Institute (MNI) 152 stereotactic space (1 mm^3^ isotropic) with linear and nonlinear registrations; (5) the brain area was categorized into gray matter, white matter, and cerebrospinal fluid; (6) individual participant tissue segmentations were constrained by tissue priors from the standard space provided with FSL.

### Functional Preprocessing

Functional preprocessing included the following steps: (1) the first 10 time points were removed; (2) slice-time correction was performed; (3) images were de-obliqued, (4) images were reoriented into right-to-left, posterior-to-anterior, inferior-to-superior orientation; (5) motion correction was performed to averaged images to obtain motion parameters; (6) skull stripping was performed; (7) global mean intensity was normalized to 10,000; (8) functional images were registered to anatomical space using linear transformation, white-matter boundary-based transformation, and the prior white-matter tissue segmentation from FSL; (8) motion artifacts were removed using ICA-AROMA with partial component regression (19); and (9) nuisance signal regression was applied, including (a) mean values from the signal in the white matter and cerebrospinal fluid derived from the prior tissue segmentations transformed from anatomical to functional space, (b) motion parameters (six head-motion parameters, six head-motion parameters from one time point before, and the 12 corresponding squared items), (c) linear trends, and (d) global signal only for one set of strategies.

### Computation of Amplitude of Low-Frequency Fluctuations

The time series for each voxel was filtered between 0.01 and 0.1 Hz, transformed into the frequency domain by fast Fourier transform and then the square root was calculated for each frequency of the power spectrum, and the averaged square root (i.e., ALFF) was obtained for each voxel. The previous anatomical-to-standard-space registration was applied to ALFF images to transform them into standard space. Smoothing (full width half maximum [fwhm] = 6 mm) and Z-score standardization were applied to registered ALFF images.

### FC Analysis

The regions of interest were selected as seeds for whole-brain FC analysis from the significant results of ALFF images from comparison of 23 patients and healthy matched controls. The FC for each voxel was defined as the Pearson's correlation between the time series within that voxel and the averaged times series in the seed. Then, FC images were registered to standard space. Smoothing (fwhm = 6) and Fisher-Z transformation were applied to the registered FC images.

### Statistical Analysis

SPSS (version 17.0; SPSS Inc., Chicago, IL, USA) was used to analyze data. A two-sample *t* test was conducted to assess differences in age, education level, clinical data, and neuropsychological assessment using intergroup comparisons. Categorical data were compared using the two-tailed chi-square test. A paired-sample *t* test was used to evaluate cognitive differences pre- and post-operation in the 15 patients.

Analysis of variance was applied for the analysis of ALFF and FC images of 23 patients, healthy matched controls, and 15 postoperative patients, using statistical tools in DPABI (20). Scheffe's multiple comparison correction was used for *post hoc* analysis, and Gaussian random field multiple comparison correction with *p* values (voxel-wise *p* < 0.001 and cluster-wise *p* < 0.025 for each tail) was applied for pairwise analysis. The whole analysis was accelerated and simplified through the cloud platform mentioned above.

A paired-sample *t* test was applied to analyze ALFF images and whole-brain FC images of the postoperative 15 patients compared to their preoperative status, using statistical tools in DPABI. Gaussian random field multiple comparison correction with *p* values (voxel-wise *p* < 0.01 and cluster-wise *p* < 0.025 for each tail) was applied for analysis. The whole analysis was accelerated and simplified through the cloud platform mentioned above.

Relationships between ALFF values of 23 patients in the significantly altered brain regions and their clinical data were analyzed with Pearson's correlation analysis. Furthermore, the brain regions that revealed significant differences in ALFF in pre- and postoperative comparisons were selected as a mask, and we extracted the mean ALFFs for each patient in these masks. We calculated the ΔALFF and Δcognitive assessment score, which represented the changes in ALFF and neuropsychological tests, respectively, before and after TKA. Then, Pearson's correlation analysis was used to study the relationship between ΔALFF in these brain regions and Δcognitive assessment score. The statistical threshold was set at a *p* value of < 0.05 to be a significant difference.

## Results

### Demographics and Clinical Data

Thirty patients and 26 controls were recruited. One patient with KOA was excluded from the research due to liver cancer as revealed by presurgical medical examination; one patient with KOA was excluded due to meningioma as revealed by MRI. Three healthy controls and five patients with KOA were excluded due to unqualified imaging, resulting from poor scanning compliance. Finally, we enrolled 23 patients (mean age: 71.2 ± 4.2 years, 15 women) and 23 age-, gender-, and education-matched controls (mean age: 71.4 ± 4.1 years, 14 women) in this study. Due to 2 patients refusing surgery and 6 patients dropping out from the research, 15 patients ultimately underwent surgery and postoperative neuropsychological assessment and MRI scan (Figure 1). The demographic and clinical data are shown in Table 1. Compared with controls, no significant demographic differences in any variables were found in 23 patients and 15 postoperative patients, including age, gender, and education years (all *p*s > 0.05). Based on the physical function scores of the WOMAC (mean score: 34.9 ± 10.7), all KOA patients experienced motor functional impairment due to KOA (21). Although pain intensity was a little lower in 15 patients postoperatively, it did not show statistical difference in pain levels compared with their presurgical status when MRI scanning (*p* > 0.05).

**Table 1:** Demographics and clinical data of controls and patients.

**Parameter**	**Healthy controls (*n* = 23)**	**KOA patients (*n* = 23)**	**Pre-TKA (*n* = 15)**	**Post-TKA (*n* = 15)**	***P*** **value**		
					**KOA patients/Healthy controls**	**Post-TKA/Healthy controls**	**Pre/Post**
Sex (M/F)	9/14	8/15	5/10	5/10	0.760^*^	0.717^*^	
Age (years)	71.4 ± 4.1	71.2 ± 4.2	70.8 ± 3.9	70.8 ± 3.9	0.851^†^	0.662^†^	
Education (years)	10.3 ± 3.4	10.2 ± 3.4	9.9 ± 3.7	9.9 ± 3.7	0.963^†^	0.783^†^	
Pain intensity					KOA patients/Post		
NRS score		3.2 ± 1.9	3.6 ± 1.5	3.4 ± 2.1	0.731^†^		0.641^#^
Physical function scores^a^ of WOMAC		34.9 ± 10.7	36.1 ± 9.7				

*KOA, knee osteoarthritis; TKA, total knee arthroplasty. WOMAC, Western Ontario and McMaster Universities Osteoarthritis Index*.

a *Scores assessed with the self-administered questionnaire in Chinese based on an English version of the WOMAC*.

* Chi-square test;

† Two-sample t test;

# *Paired two-sample t test; NRS, 0–10 pain score acquired during resting state fMRI scan*.

### Neuropsychological Assessment

There was no difference in the basic cognitive status (MoCA) in 23 patients and 15 postoperative patients compared to the controls. Compared with the controls, all AVLT tests were significantly lower in 23 patients, and only long-term recall of AVLT was significantly lower after surgery in 15 patients (*p* < 0.05). However, there were no differences in other neuropsychological tests (*p* > 0.05). The paired two-sample *t* test revealed that no significant difference was found in terms of neuropsychological assessment although the results were better after surgery when comparing the pre- and post-surgical results of the 15 patients who underwent TKA (Supplementary Table 1; *p* > 0.05).

### ALFF

**Figure 2:**
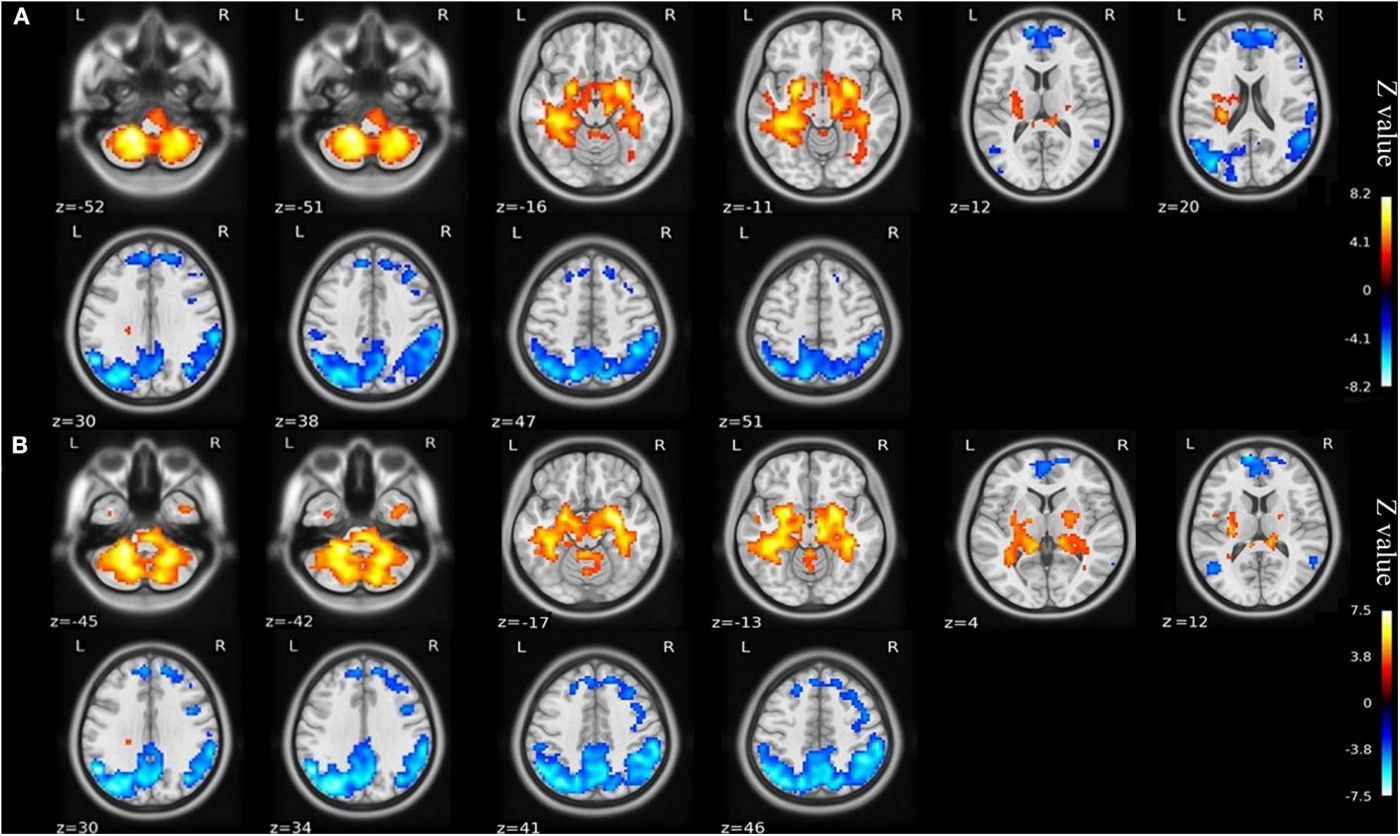
**(A)** ALFF maps show differences between KOA older patients (*n* = 23) and healthy matched controls (*n* = 23). **(B)** ALFF maps show differences between postoperative patients (*n* = 15) and healthy matched controls (*n* = 23) (voxel-wise *p* < 0.001 and cluster-wise *p* < 0.025 for each tail, corrected by GRF). Diffused ALFF decreases in the bilateral angular, precuneus, and medial superior frontal gyrus and increases in the bilateral amygdaloid nucleus and cerebellum posterior lobe (left Cerebelum_9 and right Cerebelum_8) are observed consistently in the KOA older patients and postoperative patients compared to controls. The symmetric color bars range from -vmax to vmax, which denotes the absolute value of the extreme z-value. ALFF, amplitude of low frequency fluctuation; KOA, knee osteoarthritis.

**Figure 3:**
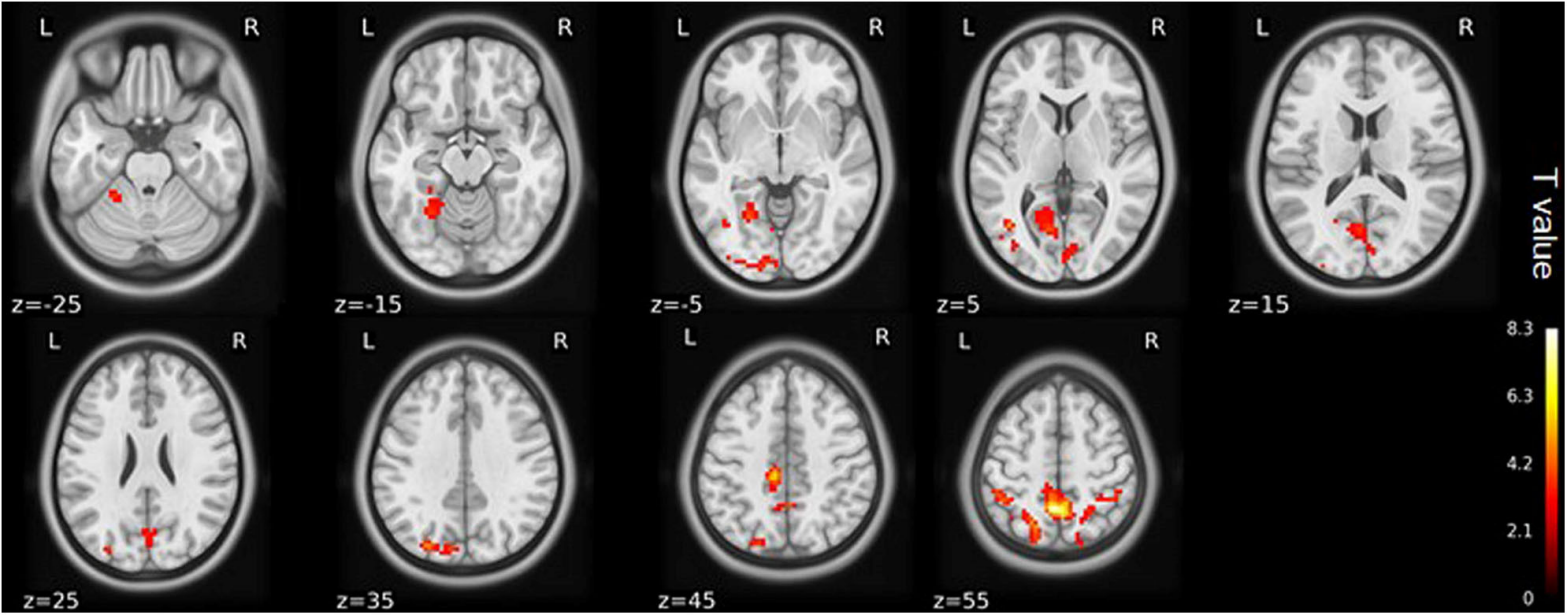
ALFF maps show differences between pre- and post-TKA patients, *n* = 15 (voxel-wise *p* < 0.01 and cluster-wise *p* < 0.025 for each tail, corrected by GRF). Paired-sample *t*-tests reveal ALFF increases in the left precuneus gyrus and middle temporal gyrus before TKA. The color bars range from zero to vmax which denotes the maximum *t*-value. ALFF, amplitude of low frequency fluctuation; TKA, total knee arthroplasty.

Compared with the healthy matched controls, 23 KOA patients had significantly decreased ALFF in the bilateral angular, precuneus, and medial superior frontal gyrus and significantly increased ALFF in the bilateral amygdaloid nucleus and cerebellum posterior lobe (left Cerebelum_9 and right Cerebelum_8). Decreased and increased ALFF were found in the same brain regions at day 7 after TKA in the 15 postoperative patients (Figure 2, Table 2). Compared with preoperative status, 15 of those patients demonstrated significantly decreased ALFF in the left precuneus gyrus and middle temporal gyrus (Figure 3, Table 3).

**Table 2:** Regions showing significant differences in ALFF between older patients with KOA and postoperative patients in comparison with healthy controls.

**Cluster name**	**Brain region**	**Cluster size**	**MNI coordinates**			**Z value^**b**^**
			**x^**a**^**	**y^**a**^**	**z^**a**^**	
**KOA patients (*****n*** **=** **23) vs. healthy controls (*****n*** **=** **23)**						
Cluster 1		4512				
	Left precuneus gyrus		−11	−66	51	−5.80
	Right precuneus gyrus		12	−76	47	−5.82
	Left angular gyrus		−54	−60	30	−7.47
	Right angular gyrus		53	−54	38	−5.42
Cluster 2		1244				
	Left medial superior frontal gyrus		−12	63	12	−5.56
	Right medial superior frontal gyrus		13	59	20	−5.06
Cluster 3		8682				
	Left cerebelum_9		−18	−51	−51	8.21
	Right cerebelum_8		28	−53	−52	6.96
	Left amygdala		−26	0	−11	6.60
	Right amygdala		25	4	−16	7.29
**Postoperative patients (*****n*** **=** **15) vs. healthy controls (*****n*** **=** **23)**						
Cluster 4		5241				
	Left precuneus gyrus		−7	−71	41	−5.70
	Right precuneus gyrus		8	−63	46	−5.63
	Left angular gyrus		−54	−60	30	−7.53
	Right angular gyrus		54	−60	34	−5.29
Cluster 5		1310				
	Left medial superior frontal gyrus		−9	63	12	−5.43
	Right medial superior frontal gyrus		13	62	4	−4.31
Cluster 6		8591				
	Left cerebelum_9		−21	−42	−45	7.36
	Right cerebelum_8		24	−54	−42	6.82
	Left amygdala		−24	1	−13	6.38
	Right amygdala		25	1	−17	6.70

*ALFF, amplitude of low frequency fluctuation; KOA, knee osteoarthritis. The results were considered significant at p < 0.025 (two tailed, voxel-wise p < 0.001, GRF corrected)*.

a *Coordinates indicate location of maximum Z-scores for clusters or location of local maxima*.

b *Z value: Maximum Z value of cluster or Z statistic of local maxima*.

**Table 3:** Regions showing significant differences in ALFF between pre- and post-TKA in older patients with KOA (*n* = 15).

**Cluster name**	**Brain region**	**Cluster size**	**MNI coordinates**			**T value^**b**^**
			**x^**a**^**	**y^**a**^**	**z^**a**^**	
Cluster 1	Left precuneus gyrus	744	0	−51	51	8.35
Cluster 2	Left middle temporal gyrus	515	−42	−66	6	5.15

*ALFF, amplitude of low frequency fluctuation; KOA, knee osteoarthritis. The results were considered significant at p < 0.025 (two tailed, voxel-wise p < 0.01, GRF corrected)*.

a *Coordinates indicate location of maximum T-value for clusters or location of local maxima*.

b *T value: Maximum T value of cluster or T statistic of local maxima*.

### Disordered Brain FC in Older Patients With KOA

**Figure 4:**
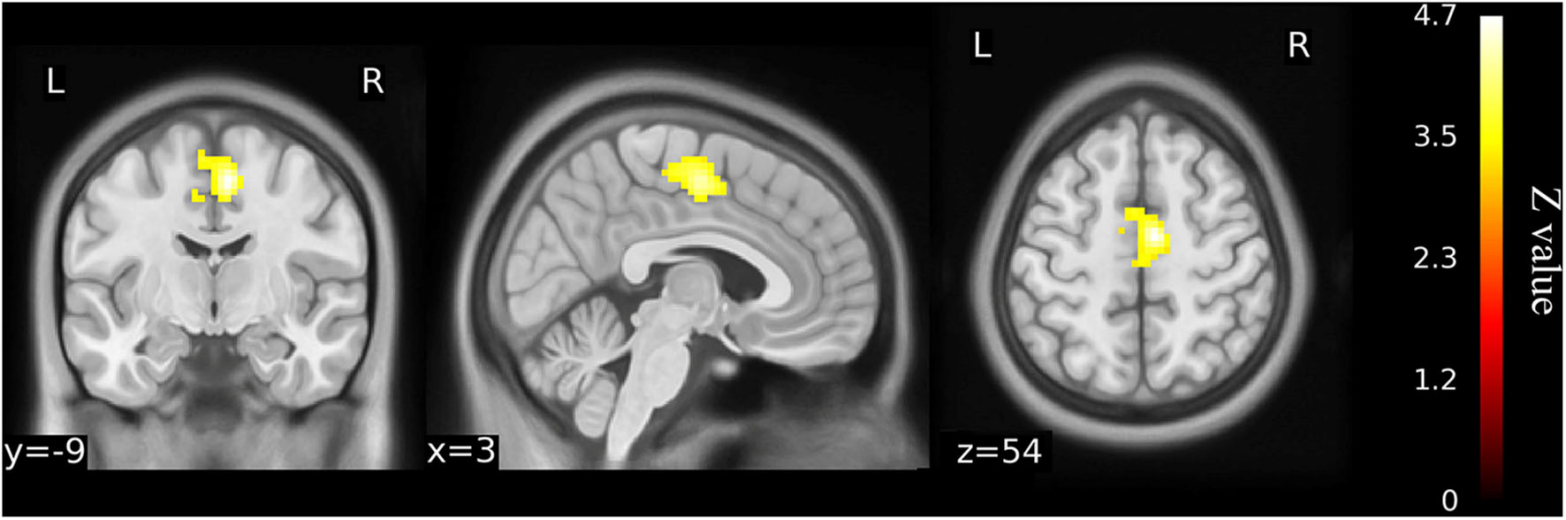
Functional connectivity shows the difference between older KOA patients and healthy matched controls (*n* = 23) on the basis of left precuneus gyrus as the ROI for analysis (voxel-wise *p* < 0.001 and cluster-wise *p* < 0.025 for each tail, corrected by GRF). Compared with controls, left precuneus gyrus has increased functional connectivity with right supplementary motor area (SMA) in the older KOA patients, but it reverts to no significant difference in the postoperative patients. The color bars range from zero to vmax, which denotes the maximum z-value. KOA, knee osteoarthritis.

**Figure 5:**
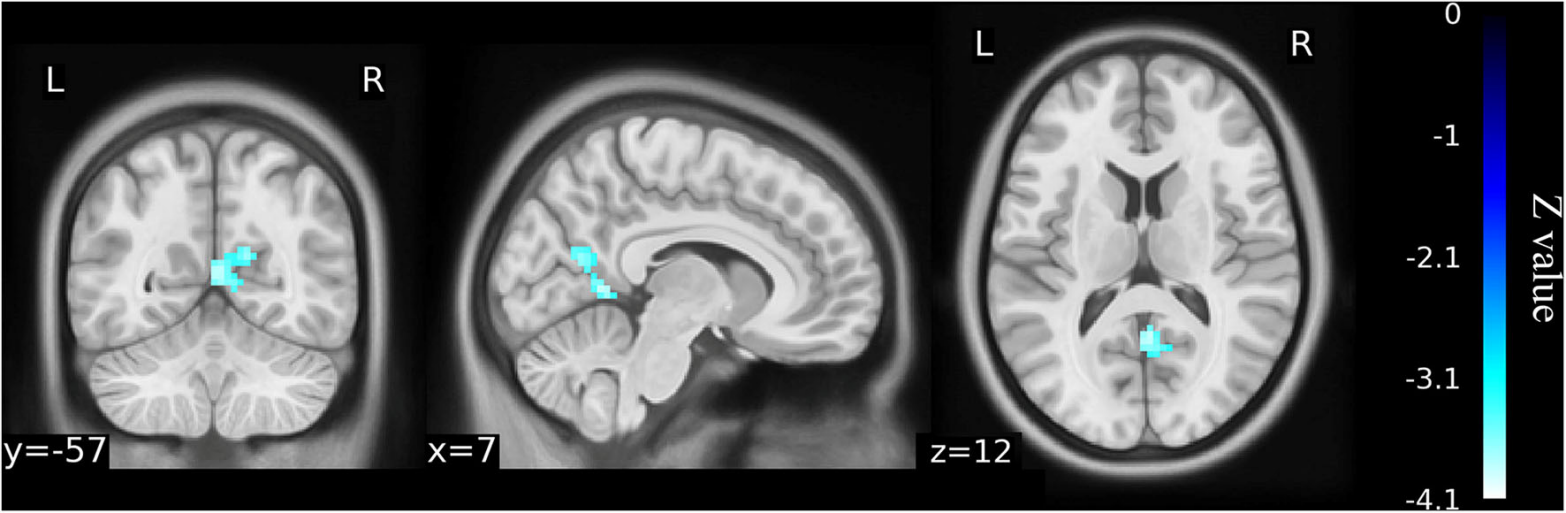
Functional connectivity shows the difference between the postoperative patients (*n* = 15) and healthy matched controls (*n* = 23) on the basis of left Cerebelum_9 as the ROI for analysis (voxel-wise *p* < 0.001 and cluster-wise *p* < 0.025 for each tail, corrected by GRF). Compared with controls, decreased functional connectivity was identified in left Cerebelum_9 with right precuneus gyrus in the postoperative patients although this was not significantly different from the older KOA patients before surgery. The color bars range from zero to –vmax, which denotes the minimum z-value. KOA, knee osteoarthritis.

When the left precuneus gyrus and left Cerebelum_9 (cerebellum posterior lobe) were selected as the regions of interest for seed-based connectivity analysis, *post hoc* analysis revealed FC differences when comparing the 23 KOA patients and 15 postoperative patients to the healthy matched controls (Figures 4, 5 and Supplementary Table 2). Although 23 KOA patients had increased FC between the left precuneus gyrus and the right supplementary motor area (SMA), this reverted to no significant difference after surgery in the 15 postoperative patients. Moreover, a decreased FC was identified in left Cerebelum_9 with right precuneus gyrus in the 15 postsurgical patients although this was not significantly different in 23 KOA patients before surgery.

As for paired-sample analysis in seed-based connectivity analysis, no differences in the 15 patients between the pre- and post-operative status were found based on those two regions of interest.

### Correlation Analysis

No relationships were found between ALFF value in the altered brain regions and clinical data, such as physical functional scores. Further, the Pearson's correlation analysis of the association between the changes of neuropsychological data, and ALFF of the significantly altered brain regions before and after TKA in the 15 patients revealed no significant correlations between ΔALFF and Δcognitive assessment score (Supplementary Table 3; *p* > 0.05).

## Discussion

This study was the first to simultaneously investigate low-frequency brain oscillations and FC alterations in older KOA patients before and at the early stage after TKA and to determine the relationship between these regional alterations and the changes of neuropsychological assessments. In this rs-fMRI study, the following main findings were revealed: (1) Brain low-frequency oscillations were decreased in the DMN and increased in the bilateral amygdaloid and cerebellum posterior lobe in the older patients with KOA compared with healthy matched controls. (2) One week after TKA, altered ALFF persisted in the same brain regions compared to controls. (3) Significantly decreased ALFF in the left precuneus gyrus and new-onset decline in the left middle temporal gyrus were identified in a paired comparison of post- and presurgical status. (4) Disordered FC was recovered, but a newly decreased FC after TKA was detected compared to controls, and no FC differences were found between pre- and post-operation. (5) No relationships were found between ALFF changes in significantly altered brain regions and the changes of neuropsychological assessment. These findings suggest that, in older patients with end-stage KOA, brain functional abnormalities were detected with the most abnormalities persisting in the same brain regions, and further low-frequency oscillation changes in specific regions were observed at the early stage after joint replacement. The ALFF may have more potential value for the detection of brain changes after TKA prior to FC and neuropsychological assessment.

Many algorithms have been developed to analyze fMRI data, such as independent component analysis, seed correlation analysis, regional homogeneity, and ALFF (17, 22, 23), and different methods of analysis can account for different aspects of integrated human brain function. Although the exact biologic mechanisms of ALFF are still unclear, it has been widely used in studies for neuropsychological diseases (24–26) due to the fact that altered ALFF is directly related to abnormal regional neuronal activity (26, 27). Similar algorithms have been applied to detect the influence of moxibustion on functional brain alterations in patients with KOA between pre- and post-treatment (28). Thus, ALFF is potentially valuable for uncovering the mechanism underlying the changes in brain function in the older patients with KOA before and after TKA. In this study, abnormal ALFF in the older patients with KOA indicated local neural dysfunction in specific brain regions, including bilateral precuneus gyrus, angular gyrus, medial superior frontal gyrus, amygdala, and cerebellum posterior lobe, which were in line with similar data in the similar regions found in previous studies (14, 15, 29).

The precuneus gyrus is a key part of the DMN and primarily involved in highly integrated work, including episodic memory retrieval, visuospatial attention, and self-processing operations (30). The angular gyrus is another important brain region of the inferior parietal lobule, representing a major posterior component of the DMN, which may participate in memory function (31). Impairment of the angular gyrus is highly correlated with damage of multiple cognitive domains involved in identifying direction and the presence of alexia, agraphia, and dyscalculia (32). Furthermore, the medial superior frontal gyrus, also in DMN, plays a key role in cognitive control and emotional regulation (33). In the present study, the cerebellum posterior lobe in our older patients was found to exhibit an increased ALFF compared with that in controls, which may indicate a functional process occurring to compensate for the decreased ALFF in the DMN. This is in line with the findings of a previous study in AD (34). This study also found, in the older KOA patients, the increased ALFF occurred in the bilateral amygdaloid nucleus, which is part of the subcortical limbic system and involved in the regulation of emotional behaviors, self-help activities, endocrine integration, and psychiatric diseases (35). The increased spontaneous activity in this nucleus has been reported in the context of some mental disorders (36). Furthermore, our data suggest that regional low-frequency oscillations of this region are sensitive to chronic pain, which are consistent with previous studies (15, 37). Overall, in older patients with KOA, altered ALFF in these important brain regions may contribute to the prevalence of memory function decline from neuropsychological assessment in this study and chronic pain in previous studies (38–41).

Previous studies report that the prevalence of cognitive decline 1 week after TKA in the older patients was not low (5, 6) although the mechanism remains unclear. In our study, low-frequency brain oscillations of these patients after TKA showed the abnormalities of spontaneous brain activity persisted in the same regions, which was in line with memory function decline in neuropsychological assessment compared with the controls. The reasons for this persistence might be multifactorial. Peripheral inflammation may be still sustainable at the early stage after TKA due to local tissue injury by surgery and “foreign” material stimuli by prosthesis implantation. Furthermore, similar with preoperative chronic pain, postoperative pain intensity revealed no significant improvement given that all patients need early postoperative rehabilitation, which could yield positive effects on memory decline in neuropsychological testing after TKA compared to the controls. By using the paired-sample analysis, which excludes any potential confounding effects of different sample sizes before and after surgery, the comparison of pre- and postoperative ALFF revealed a further decrease in the left precuneus gyrus and middle temporal gyrus after surgery. Particularly, a significantly decreased ALFF in the left precuneus gyrus suggests that abnormal regional neuronal activity exacerbated postoperatively. In contrast to the healthy matched controls, all KOA patients and postoperative patients did not show abnormal ALFF in the left middle temporal gyrus. This region is suggested to be closely related to cognitive processing of language (42, 43). Thus, we speculate that the decreased ALFF we observed in this area might imply the new-onset language function decline although no related result was found in the cognitive assessment. Multiple factors may contribute to these changes in ALFF, such as pain, anesthesia, surgery-induced trauma, and medications taken after surgery (44). Given that we found no difference in pain intensity between pre-and post-operative status by paired-sample analysis, it is hard to conclude that pain is a major factor for these changes. Previous studies involving rs-fMRI analyses suggest that general anesthetics, such as propofol, midazolam, and inhaled anesthetics, can affect brain function (45–47), especially for regional brain activity (48, 49). In the present study, we did not administer any sedatives or general anesthetics during surgery, and local anesthetics without opioids were solely used for spinal anesthesia; therefore, the effects of general anesthetics on brain function can be negligible. A high risk of mild cognitive impairment and dementia is usually increased with age (50), which can be synergistically enhanced with potential neuronal insults from stress and central neuroinflammation caused by surgery and surgical trauma (51, 52). Practically, it is difficult to exclude these confounders, but comparing ALFF changes between the pre- and post-TKA surgery would be helpful to identify their contributions *per se* and worth investigating in future study.

The use of a single analytical method for rs-fMRI might mean that some important information is missed. Therefore, we analyzed FC, which defines the temporal correlation of the blood oxygen level-dependent signals of distinct brain areas to reflect the information integration between brain areas (53). The regions of interest were selected to include regions in which the difference in ALFF images between all the KOA patients and the healthy matched controls were most significant. Comparing with controls revealed that functional connectivity between the left precuneus gyrus and right SMA was increased in all KOA patients before surgery, but no difference was found in the postoperative patients at the early stage after TKA, which meant adaptation in brain functioning for patients' altered motor performance. The SMA plays a key role in movement planning and initiation, especially in self-initiated movements (54). The increased FC in the SMA may be a compensatory effect for poor motor function of the KOA patients, which was similar to previous studies that demonstrated that the activation of SMA may be caused by the disease itself or by patients' altered motor functions (15, 37, 55). This indicates that knee arthroplasty can improve joint mobility and may contribute to the recovery of disordered brain function. However, another remarkable finding of the present study was that knee arthroplasty induced significant decreases in the FC between the left cerebelum_9 (belonging to cerebellum posterior lobe) and the right precuneus gyrus. The function of these two important brain regions should be studied more due to their abnormalities in the regional spontaneous activity and mutual FC decline at the same time postoperatively compared to the healthy matched controls. There are multiple lines of evidence suggesting a cognitive role for the cerebellum, and previous studies suggest that the cerebellum, especially the posterior cerebellar regions, has evolved in a network-specific fashion to mediate cognition and emotion (56). In line with the increased ALFF in the posterior cerebellar regions that was observed in the present study, this increased activation suggests that compensatory mechanisms exist in some diseases involving cognitive decline (34, 57). The cerebellum is linked via feedforward projections to the cerebral sensorimotor regions that subserve higher-order cognitive functions in the prefrontal and parietal cortices and the cingulate and parahippocampal gyri. A loss of cerebellar contribution to the cerebrocerebellar circuits may cause cognitive impairment in executive function, working memory, and visual spatial cognition (58). Furthermore, the precuneus gyrus, one of the functional centers, is a crucial hub of the DMN. Such disordered functional connectivities between the hub of DMN and other regions can be commonly regarded as the fundamental basis for cognitive dysfunction (59). However, our research reveals that no differences between the pre-and post-operative status in older patients with KOA were found in FC using paired-sample analysis, which indicates that the changes in FC might occur later than the alterations of low-frequency brain oscillations.

In this study, some slight improvements of cognition were found after surgery through a comparison of all KOA and postoperative patients to healthy matched controls and a comparison of patients in pre- and post-surgical status. Such performance improving in neuropsychological testing can be attributable to a learning or practice effect, especially for repeated testing at only a 1-week interval (60). A few studies report on such relationships between the ALFF changes in specific regions of brain and changes in neuropsychological assessment, and our study is unable to address the contention that functional alterations in individual brain regions are related to parameters in neuropsychological test. It has been suggested that rs-fMRI is more sensitive for detecting early functional brain changes and can identify changes earlier than behavioral tests and clinical symptoms for assessing cognitive function in older patients (61, 62). Therefore, rs-fMRI analysis may be more valuable than neuropsychological tests to detect cognitive decline in older people with KOA ready for TKA.

The current research has some limitations. First, it is difficult to fully exclude confounders of pre-and post-TKA on the ALFF/FC changes in the present study, such as the surgical trauma, pain, and medications taken after surgery. Therefore, a strict control group or randomized controlled trial–type study would be helpful to rule out the exact causes of these changes in the future. Second, our results, particularly the correlation between altered brain function and cognitive changes, were derived from a relative small sample size, and therefore, the robustness of this correlation is not known. Third, long-term follow-up was not done, and therefore, brain function changes after TKA in the long term is not known. Fourth, the participants in the healthy control group were only tested once. Considering that the neurological status of the healthy controls was almost unchanged over months, we did not carry out second MRI examinations in this group. In addition, some patients, particularly if they experienced no discomfort after surgery, were not compliant with follow-up scans. Similarly, it was difficult to obtain second scans for healthy volunteers in the short research period. That made the different sample sizes before and after surgery, especially for postoperative patients, which might yield result bias.

## Conclusions

In older patients with end-stage KOA, low-frequency brain oscillations revealed a decrease in the DMN and an increase in the amygdala and the posterior lobe of the cerebellum with persistence in the same brain regions and further changes in left precuneus gyrus and middle temporal gyrus observed at the early stage after knee replacement. Although disordered FC backed to no difference, a newly disturbed connectivity occurred after joint arthroplasty. Despite coincident alterations in brain function and cognitive assessment, there did not appear to be any association between the two, which may be very likely due to low power of small sizes in our study. Nevertheless, our data demonstrated that rs-fMRI with the ALFF and FC algorithm may have practical value for detecting brain function changes superior to neuropsychological assessment and may be useful to uncover the mechanism of cognitive decline before and at the early stage after TKA in older patients with KOA.

## Data Availability Statement

The original contributions presented in the study are included in the article/Supplementary Materials, further inquiries can be directed to the corresponding author/s.

## Ethics Statement

The studies involving human participants were reviewed and approved by Institutional Review Board of Xuanwu Hospital, Capital Medical University, Code: 2018-047. The patients/participants provided their written informed consent to participate in this study.

## Author Contributions

FL, ZGQ, DQM, and TLW contributed conception and design of the study. FYL and MD organized the database. ZGQ performed the statistical analysis. FL and GWL wrote the first draft of the manuscript. GLC, ZL, WX, and HQF wrote sections of the manuscript. All authors contributed to manuscript revision, read, and approved the submitted version.

## Conflict of Interest

The authors declare that the research was conducted in the absence of any commercial or financial relationships that could be construed as a potential conflict of interest.

## Abbreviations

ALFF: amplitude of low-frequency fluctuation
CDT: clock drawing test
DMN: default mode network
FC: functional connectivity
SMA: supplementary motor area
TKA: total knee arthroplasty.
