# Supplementary material for: Altered Intrinsic Brain Activity and Functional Connectivity Before and After Knee Arthroplasty in the Elderly: A Resting-State fMRI Study
Source: Front Neurol. 2020 Sep 29;11:556028. doi: 10.3389/fneur.2020.556028 (PMC7550714; doi:10.3389/fneur.2020.556028)
Supplement: Supplementary file 1 [file Data_Sheet_1.ZIP › supplemental data3.docx]

**Article Title**

Data of Correlations between the changes in neuropsychological tests and amplitude of low frequency fluctuation changes in brain regions before and after knee arthroplasty for 15 postoperative patients.

**Authors**

Fei Lan^1^, Guanwen Lin^1^, Guanglei Cao^2^, Zheng Li^2^, Fangyan Liu^1^, Mei Duan^1^, Huiqun Fu^1^, Wei Xiao^1^, Daqing Ma^3^, Zhigang Qi^4^, Tianlong Wang^1^

**Affiliations**

^1^Department of Anesthesiology Xuanwu Hospital, Capital Medical University, National Clinical Research Center for Geriatric Disorders, Beijing Institute for Brain Disorders, No.45, Changchun Street, Beijing 100053, China

^2^Department of Orthopedics, Xuanwu Hospital, Capital Medical University, Beijing, China

^3^Anaesthesia Research of the Section of Anaesthetics, Pain Medicine & Intensive Care, Department of Surgery & Cancer, Faculty of Medicine, Imperial College London, and Chelsea and Westminster Hospital, London, UK

^4^Department of Radiology, Xuanwu Hospital, Capital Medical University, Beijing, China

Corresponding author(s)

Prof. Tianlong Wang(w_tl5595@hotmail.com )

Dr. Zhigang Qi (qizhigang2007@163.com)

**Abstract**

The brain regions that revealed significant differences in amplitude of low frequency fluctuation (ALFF) in pre- and postoperative comparisons were selected as a mask, and extracted the mean ALFFs for each patient in these masks. We calculated the ΔALFF and Δcognitive assessment score, which represented the changes in ALFF and neuropsychological tests, respectively, before and after knee replacement. Then, Pearson’s correlation analysis was used to study the relationship between ΔALFF in these brain regions and Δcognitive assessment score. The statistical threshold was set at a p value of <0.05 to be a significant difference

**Keywords**

amplitude of low-frequency fluctuation; neuropsychological test ; relationship; knee replacement

**Specifications Table**

| **Subject** | Radiology and Imaging; Cognitive Neuroscience |
| --- | --- |
| **Specific subject area** | relationship between the changes of ALFF in significantly altered brain regions and the changes of cognitive assessment score |
| **Type of data** | Table |
| **How data were acquired** | the data were acquired by MRI scan and survey  the Instruments: analysis software |
| **Data format** | Raw  Analyzed |
| **Parameters for data collection** | The brain regions that revealed significant differences in ALFF in pre- and postoperative comparisons were selected as a mask, and extracted the mean ALFFs for each patient in these masks. All participants received the cognitive scale cooperatively. |
| **Description of data collection** | rs-fMRI images were generated using a rapid-gradient echo-planar imaging sequence (239 volumes, repetition time = 2,000 ms, echo time = 40 ms, field of view = 240 × 240 mm2, flip angle = 90°, section thickness = 4 mm, acquisition matrix = 64 × 64, a total of 28 slices covering the whole brain). Three-dimensional T1-weighted magnetization-prepared rapid-gradient echo sagittal images were collected using the following parameters: repetition time = 1900 ms, echo time = 2.2 ms, inversion time (TI) = 900 ms, FA = 9°, resolution = 256 × 256 matrix, a total of 176 slices with a thickness of 1.0 mm, and voxel size = 1 × 1 × 1 mm. Detailed neuropsychological tests were carried out by the same neuropsychologist |
| **Data source location** | Institution:Department of Anesthesiology, Xuanwu Hospital, Capital Medical University  City/Town/Region: Beijing  Country: China |
| **Data accessibility** | With the article |

**Value of the Data**

- Important supplemental data for correlation analysis betweenΔALFF andΔcognitive assessment score in manuscript
- Indicating the inconsistency between the changes in ALFF and neuropsychological assessment
- Being a pilot result for longitudinal study in future

**Supplemental table 3. Correlations between the changes in neuropsychological tests and ALFF changes in brain regions before and after** **knee arthroplasty for 15 postoperative patients.**

The results indicated that there were no correlations between the changes in neuropsychological tests and ALFF changes in brain regions. A. U. = arbitrary unit, ALFF 1 or 2 = amplitude of low-frequency fluctuation in the left precuneus gyrus or left middle temporal gyrus, Δrepresents differences before and after knee arthroplasty for 15 older patients in Post group. Values presented are the correlation coefﬁcient(P values). VFT = Verbal Fluency Test, AVLT = Auditory Verbal Learning Test(short term and long delayed term recall), STT-B = Shape trails test, CDT = clock drawing test(30 scores in total).

|  | **ΔALFF 1( A. U.)** | **ΔALFF 2( A. U.)** |
| --- | --- | --- |
| ΔALVT-S(score) | -0.15(0.60) | -0.01(0.98) |
| ΔAVLT-L(score) | 0.24(0.40) | 0.03(0.91) |
| ΔVFT(score) | -0.12(0.67) | -0.34(0.22) |
| ΔSTT-B(second) | -0.05(0.85) | -0.20(0.48) |
| ΔCDT30(score) | -0.42(0.12) | -0.10(0.72) |

**Data Description**

The Pearson’s correlation analysis of the association between the changes of neuropsychological data and ALFF of the significantly altered brain regions before and after knee replacement in the 15 patients revealed no significant correlations between ΔALFF and Δcognitive assessment score (Supplemental table 3; p > 0.05).

**Experimental Design, Materials, and Methods**

The brain regions that revealed significant differences in amplitude of low frequency fluctuation (ALFF) in pre- and postoperative comparisons were selected as a mask, and extracted the mean ALFFs for each patient in these masks. We calculated the ΔALFF and Δcognitive assessment score, which represented the changes in ALFF and neuropsychological tests, respectively, before and after knee replacement. Then, Pearson’s correlation analysis was used to study the relationship between ΔALFF in these brain regions and Δcognitive assessment score. The statistical threshold was set at a p value of <0.05 to be a significant difference

**Acknowledgments**

None

**Competing Interests**

The authors declare that they have no known competing financial interests or personal relationships which have, or could be perceived to have, influenced the work reported in this article.

**References**
