# Supplementary material for: Altered Intrinsic Brain Activity and Functional Connectivity Before and After Knee Arthroplasty in the Elderly: A Resting-State fMRI Study
Source: Front Neurol. 2020 Sep 29;11:556028. doi: 10.3389/fneur.2020.556028 (PMC7550714; doi:10.3389/fneur.2020.556028)
Supplement: Supplementary file 1 [file Data_Sheet_1.ZIP › upplemental data1.docx]

**Article Title**

Data of Neuropsychological assessment

**Authors**

Fei Lan^1^, Guanwen Lin^1^, Guanglei Cao^2^, Zheng Li^2^, Fangyan Liu^1^, Mei Duan^1^, Huiqun Fu^1^, Wei Xiao^1^, Daqing Ma^3^, Zhigang Qi^4^, Tianlong Wang^1^

**Affiliations**

^1^Department of Anesthesiology Xuanwu Hospital, Capital Medical University, National Clinical Research Center for Geriatric Disorders, Beijing Institute for Brain Disorders, No.45, Changchun Street, Beijing 100053, China

2Department of Orthopedics, Xuanwu Hospital, Capital Medical University, Beijing, China

3Anaesthesia Research of the Section of Anaesthetics, Pain Medicine & Intensive Care, Department of Surgery & Cancer, Faculty of Medicine, Imperial College London, and Chelsea and Westminster Hospital, London, UK

4Department of Radiology, Xuanwu Hospital, Capital Medical University, Beijing, China

Corresponding author(s)

Prof. Tianlong Wang(w_tl5595@hotmail.com )

Dr. Zhigang Qi (qizhigang2007@163.com)

**Abstract**

The baseline general cognitive data were collected from all participants using the Montreal Cognitive Assessment (MoCA) system, and then detailed neuropsychological tests were carried out by the same neuropsychologist. The tests included verbal fluency (VFT), auditory verbal learning (AVLT; short-term and long-delayed-term recall), shape trails test-B (STT-B) and clock drawing test (CDT, 30 points in total). Particular attention was paid to memory and executive ability, which are commonly seen to decline after knee arthroplasty. All healthy matched controls underwent these assessment once, and all 23 patients received these assessments on the day just prior to MRI scan before surgery and due to drop off or refused surgery, 15 of those patients had the assessment at 1 week after surgery. A two-sample t test was conducted to assess differences in neuropsychological assessment using inter-group comparisons. A paired-sample t test was used to evaluate cognitive differences between pre- and post-operation in the 15 patients.

**Keywords**

Neuropsychological; MRI; knee arthroplasty; cognitive

**Specifications Table**

| **Subject** | Cognitive Neuroscience |
| --- | --- |
| **Specific subject area** | The difference of neuropsychological assessment in older patients with knee osteoarthritis and these postoperative patients compared to healthy matched controls, and the difference of neuropsychological assessment in postoperative patients compared with their preoperative status. |
| **Type of data** | Table |
| **How data were acquired** | the data were acquired by survey  the Instruments: Assessment scale |
| **Data format** | Raw  Analyzed |
| **Parameters for data collection** | All participants met the inclusion criteria with the completion of the scale cooperatively . |
| **Description of data collection** | detailed neuropsychological tests were carried out by the same neuropsychologist |
| **Data source location** | Institution:Department of Anesthesiology, Xuanwu Hospital, Capital Medical University  City/Town/Region: Beijing  Country: China |
| **Data accessibility** | With the article |

**Value of the Data**

- Important supplemental data for cognitive assessment in manuscript
- Indicating the difference between patients and healthy matched controls
- Being a pilot result for longitudinal study in future How can these data be used for further insights and development of experiments

Supplemental table 1. Neuropsychological assessment

| Parameter | Healthy controls  (n = 23) | KOA patients  (n = 23) | Pre-TKA  (n = 15) | Post-TKA  (n = 15) | P value | | |
| --- | --- | --- | --- | --- | --- | --- | --- |
|  |  |  |  |  | KOA patients  /Healthy controls | Post-TKA  / Healthy controls | Pre/Post |
| MoCA(score) | 24.5±3.1 | 24.3±3.1 | 24.0±3.4 | 24.7±3.5 | 0.909^†^ | 0.848^†^ | 0.583^#^ |
| ALVT-S(score)  AVLT-L(score)  VFT(score)  STT-B(second)  CDT30(score) | 8.5±3.2  11.5±2.3  16.2±2.6  189.5±44.8  25.9±3.0 | 6.0±1.8  5.4±2.1  16.0±2.8  187.2±45.7  25.9±2.9 | 7.6±1.5  7.3±1.5  15.9±3.2  189.7±43.0  25.6±3.0 | 7.7±2.3  7.5±1.9  16.8±4.1  169.7±47.7  26.3±3.0 | 0.002^†^  0.000^†^  0.783^†^  0.867^†^  0.996^†^ | 0.401^†^  0.000^†^  0.581^†^  0.207^†^  0.678^†^ | 0.843^#^  0.840^#^  0.360^#^  0.074^#^  0.155^#^ |

KOA = knee osteoarthritis; TKA = total knee arthroplasty. *Chi-square test; †Two-sample t test; # Paired two-sample t test; MoCA = Montreal Cognitive Assessment; VFT = Verbal Fluency Test; AVLT = Auditory Verbal Learning Test( short term and long delayed term recall ); STT-B = Shape trails test; CDT = clock drawing test( 30 scores in total).

**Data Description**

supplemental table 1 showed that there was no difference in the basic cognitive status (MoCA) in 23 patients and 15 postoperative patients compared to the controls. Compared with the controls, all AVLT tests showed significantly lower in 23 patients, and only long-term recall of AVLT showed significantly lower after surgery in 15 patients (p < 0.05). However, there were no differences in other neuropsychological test (p > 0.05). The paired two-sample t test revealed that no significant difference was found in terms of neuropsychological assessment although the results were better after surgery, when comparing the pre- and post-surgical results of the 15 patients who underwent knee arthroplasty.

**Experimental Design, Materials, and Methods**

The baseline general cognitive data were collected from all participants using the Montreal Cognitive Assessment (MoCA) system, and then detailed neuropsychological tests were carried out by the same neuropsychologist. The tests included verbal fluency (VFT), auditory verbal learning (AVLT; short-term and long-delayed-term recall), shape trails test-B (STT-B) and clock drawing test (CDT, 30 points in total). Particular attention was paid to memory and executive ability, which are commonly seen to decline after knee replacement. All healthy matched controls underwent these assessment once, and all 23 patients received these assessments on the day just prior to MRI scan before surgery and due to drop off or refused surgery, 15 of those patients had the assessment at 1 week after surgery . A two-sample t test was conducted to assess differences in neuropsychological assessment using inter-group comparisons. A paired-sample t test was used to evaluate cognitive differences between pre- and post-operation in the 15 patients.

**Acknowledgments**

None

**Competing Interests**

The authors declare that they have no known competing financial interests or personal relationships which have, or could be perceived to have, influenced the work reported in this article.

**References**
