# Supplementary material for: Altered Intrinsic Brain Activity and Functional Connectivity Before and After Knee Arthroplasty in the Elderly: A Resting-State fMRI Study
Source: Front Neurol. 2020 Sep 29;11:556028. doi: 10.3389/fneur.2020.556028 (PMC7550714; doi:10.3389/fneur.2020.556028)
Supplement: Supplementary file 1 [file Data_Sheet_1.ZIP › supplemental data2.docx]

**Article Title**

Data of regions showing significantly differences in functional connectivity between older patients with knee ostarthritis and postoperative patients in comparison with healthy controls.

**Authors**

Fei Lan^1^, Guanwen Lin^1^, Guanglei Cao^2^, Zheng Li^2^, Fangyan Liu^1^, Mei Duan^1^, Huiqun Fu^1^, Wei Xiao^1^, Daqing Ma^3^, Zhigang Qi^4^, Tianlong Wang^1^

**Affiliations**

^1^Department of Anesthesiology Xuanwu Hospital, Capital Medical University, National Clinical Research Center for Geriatric Disorders, Beijing Institute for Brain Disorders, No.45, Changchun Street, Beijing 100053, China

^2^Department of Orthopedics, Xuanwu Hospital, Capital Medical University, Beijing, China

^3^Anaesthesia Research of the Section of Anaesthetics, Pain Medicine & Intensive Care, Department of Surgery & Cancer, Faculty of Medicine, Imperial College London, and Chelsea and Westminster Hospital, London, UK

^4^Department of Radiology, Xuanwu Hospital, Capital Medical University, Beijing, China

Corresponding author(s)

Prof. Tianlong Wang(w_tl5595@hotmail.com )

Dr. Zhigang Qi (qizhigang2007@163.com)

**Abstract**

The regions of interest (Left precuneus gyrus and Left cerebelum_9) were selected as seeds for whole-brain functional connectivity analysis from the significant results of amplitude of low-frequency fluctuation images from comparison of 23 patients and healthy matched controls. The functional connectivity for each voxel was defined as the Pearson’s correlation between the time series within that voxel and the averaged times series in the seed. Then, functional connectivity images were registered to standard space. Smoothing (fwhm = 6) and Fisher-Z transformation were applied to the registered functional connectivity images. Analysis of variance was applied for the functional connectivity images of 23 patients, healthy matched controls and 15 postoperative patients, using statistical tools in DPABI [1]. Scheffe’s multiple comparison correction was used for post-hoc analysis, and Gaussian random field multiple comparison correction with p values (voxel-wise p < 0.001 and cluster-wise p < 0.025 for each tail) was applied for pairwise analysis. The whole analysis was accelerated and simplified through the cloud platform mentioned above.

A paired-sample t-test was applied to analyze whole-brain functional connectivity images of the postoperative 15 patients compared to their preoperative status, using statistical tools in DPABI. Gaussian random field multiple comparison correction with p values (voxel-wise p < 0.01 and cluster-wise p < 0.025 for each tail) was applied for analysis.

**Keywords**

functional connectivity; precuneus gyrus ; cerebelum

**Specifications Table**

| **Subject** | Radiology and Imaging |
| --- | --- |
| **Specific subject area** | The difference of functional connectivity in older patients with knee osteoarthritis and postoperative patients of these compared to healthy matched controls, and the difference of functional connectivity in postoperative patients compared with their preoperative status. |
| **Type of data** | Table |
| **How data were acquired** | the data were acquired by MRI scan  the Instruments: analysis software |
| **Data format** | Raw  Analyzed |
| **Parameters for data collection** | The functional connectivity for each voxel was defined as the Pearson’s correlation between the time series within that voxel and the averaged times series in the seed. Then, functional connectivity images were registered to standard space. Smoothing (fwhm = 6) and Fisher-Z transformation were applied to the registered functional connectivity images. |
| **Description of data collection** | rs-fMRI images were generated using a rapid-gradient echo-planar imaging sequence (239 volumes, repetition time = 2,000 ms, echo time = 40 ms, field of view = 240 × 240 mm2, flip angle = 90°, section thickness = 4 mm, acquisition matrix = 64 × 64, a total of 28 slices covering the whole brain). Three-dimensional T1-weighted magnetization-prepared rapid-gradient echo sagittal images were collected using the following parameters: repetition time = 1900 ms, echo time = 2.2 ms, inversion time (TI) = 900 ms, FA = 9°, resolution = 256 × 256 matrix, a total of 176 slices with a thickness of 1.0 mm, and voxel size = 1 × 1 × 1 mm. |
| **Data source location** | Institution:Department of Anesthesiology, Xuanwu Hospital, Capital Medical University  City/Town/Region: Beijing  Country: China |
| **Data accessibility** | With the article |

**Value of the Data**

- Important supplemental data for functional connectivity in manuscript
- Indicating the difference between patients and healthy matched controls as to functional connectivity
- Being a pilot result for longitudinal study in future

**Supplemental table 2: Regions showing significantly differences in FC between older patients with KOA and postoperative patients in comparison with healthy controls.**

|  | ROI | Brain region | MNI coordinates  x y z | Peak T value | Cluster size  (Voxels) |
| --- | --- | --- | --- | --- | --- |
| **KP vs. HCs** | Left precuneus gyrus | Right supplementary motor area | 6 -9 54 | 4.65 | 202 |
| **Post vs. HCs** | Left precuneus gyrus | None | None | None | None |
| **KP vs. HCs** | Left cerebelum_9 | None | None | None | None |
| **Post vs. HCs** | Left cerebelum_9 | Right precuneus gyrus | 15 -60 18 | -4.15 | 109 |

FC = functional connectivity; KOA = knee osteoarthritis; HCs = healthy older controls, n = 23; KP = older patients with KOA, n = 23; Post = the postoperative patients, n = 15. The results were considered significant at p<0.025(two tailed, GRF corrected, voxel-wise p<0.001). T value = the t value of two-sample t-test in the voxel showing maximum FC difference. Negative t value represents decrease, and positive t value represents increase.

**Data Description**

Supplemental table 2 showed that when the left precuneus gyrus and left Cerebelum_9 (cerebellum posterior lobe) were selected as the regions of interest for seed-based connectivity analysis, post-hoc analysis revealed functional connectivity differences when comparing the 23 patients and 15 postoperative patients to the healthy matched controls. Although 23 patients had increased functional connectivity between the left precuneus gyrus and the right supplementary motor area (SMA), this reverted to no significant difference after surgery in the 15 postoperative patients. Moreover, a decreased functional connectivity was identified in left Cerebelum_9 with right precuneus gyrus in the 15 post-surgical patients, although this was not significantly different in 23 patients before surgery.

**Experimental Design, Materials, and Methods**

The regions of interest (Left precuneus gyrus and Left cerebelum_9) were selected as seeds for whole-brain functional connectivity analysis from the significant results of amplitude of low-frequency fluctuation images from comparison of 23 patients and healthy matched controls. The functional connectivity for each voxel was defined as the Pearson’s correlation between the time series within that voxel and the averaged times series in the seed. Then, functional connectivity images were registered to standard space. Smoothing (fwhm = 6) and Fisher-Z transformation were applied to the registered functional connectivity images. Analysis of variance was applied for the functional connectivity images of 23 patients, healthy matched controls and 15 postoperative patients, using statistical tools in DPABI [1]. Scheffe’s multiple comparison correction was used for post-hoc analysis, and Gaussian random field multiple comparison correction with p values (voxel-wise p < 0.001 and cluster-wise p < 0.025 for each tail) was applied for pairwise analysis.

**Acknowledgments**

None

**Competing Interests**

The authors declare that they have no known competing financial interests or personal relationships which have, or could be perceived to have, influenced the work reported in this article.

**References**

[1] Yan, C. G., Wang, X. D., Zuo, X. N., and Zang, Y. F. (2016). DPABI: Data Processing & Analysis for (Resting-State) Brain Imaging. Neuroinformatics. 14, 339-351. doi: 10.1007/s12021-016-9299-4
